# Clinicopathological characteristics and treatment patterns of combined small-cell lung cancer: a real-world single-center study with a mini review

**DOI:** 10.3389/fimmu.2025.1652803

**Published:** 2025-09-17

**Authors:** Liu Gong, Hongsen Li, Jiawei Shou, Jin Sheng, Wei Jin, Haizhou Lou, Da Li, Hong Hu, Yong Fang

**Affiliations:** Department of Medical Oncology, Sir Run Run Shaw Hospital, Zhejiang University School of Medicine, Hangzhou, China

**Keywords:** immunotherapy, chemotherapy, combined small-cell lung cancer, real-world study, treatment strategies

## Abstract

**Background:**

Combined small-cell lung cancer is a rare subtype of SCLC, which is characterized by the coexistence of SCLC with any histological type of non-small cell lung cancer. There is limited clinical data. We aimed to explore the clinicopathological features and prognosis of C-SCLC patients who received anti-tumor therapy.

**Methods:**

Eligible patients were histopathologically confirmed adult C-SCLC who received anti-tumor treatment at Sir Run Run Hospital. This analysis aimed to describe the clinicopathological characteristics and evaluate the tumor response rate (RR), disease control rate (DCR), and progression-free survival (PFS).

**Results:**

Thirty patients were included. Eighty-three point three three percent were male, and sixty-six point six seven percent were non-smokers. Squamous cell carcinoma (SCC; 11/30) and adenocarcinoma (AC; 11/30) were the most frequently observed mixed components, followed by large-cell neuroendocrine carcinoma (LCNEC; 8/30). Patients received immunochemotherapy (13/30), platinum-based chemotherapy (9/30), or anti-EGFR-/anti-VEGF-based therapy (8/30). Most patients used the anti-PD-1 inhibitor Serplulimab (n=7). Among 27 patients with measurable disease, the RR and DCR were 51.85% (95% CI: 31.95 - 71.33%) and 85.19% (95% CI: 66.27 - 95.81%), respectively. The median PFS was 9.70 months (95% CI: 4.37 - 18.73). The median PFS of C-SCLC mixed with LCNEC was higher than that of those mixed with AC or SCC (10.62 *vs.* 9.70 *vs.* 4.17 months; *P* = 0.858); patients are more likely to benefit from immunotherapy than from chemotherapy and targeted therapy (9.70 *vs.* 5.27 *vs.* NR months; *P* = 0.685).

**Conclusion:**

Our findings provide a basis for systematic treatment strategies in C-SCLC and suggest that patients may derive benefit from immunotherapy, although further studies are needed to confirm these observations.

## 1 Introduction

Small-cell lung cancer (SCLC), constituting approximately 15% of all lung cancer cases, is characterized by its aggressive nature and notoriously poor survival rates (1). This neoplasm is strongly associated with tobacco carcinogen exposure and is marked by an exceptionally high proliferative rate and a strong predilection for early metastasis.

Combined small-cell lung cancer (C-SCLC) is a rare yet increasingly recognized subtype of SCLC initially identified by the World Health Organization (WHO) in 1981, and SCLC was subsequently stratified into pure SCLC and C-SCLC in 1999 (2). C-SCLC is defined by the coexistence of SCLC with any histological type of non-small cell lung cancer (NSCLC), including adenocarcinoma (AC), squamous-cell carcinoma (SCC), large-cell carcinoma (LCC), and large-cell neuroendocrine carcinoma (LCNEC), among other less common variants such as spindle-cell carcinoma (SpCC) or giant cell carcinoma (GCC) (3). The current iteration of the National Comprehensive Cancer Network (NCCN) for SCLC establishes a revised diagnostic threshold for C-SCLC, requiring the presence of ≥ 10% LCNEC within the tumor architecture as a histopathological prerequisite for this composite neuroendocrine tumor designation (4). The prevalence of C-SCLC has seen a rise in recent years, representing 2% - 30.1% of all SCLC cases, mainly due to inconsistencies in the types of specimens used in different study centers (5–7).

From a molecular perspective, C-SCLC demonstrates conserved genomic aberrations similar to those of conventional SCLC, exhibiting chromosomal instability coupled with an exceptionally high tumor mutational burden (TMB). Functional inactivation of *TP_53_* and *RB_1_*, common tumor suppressor genes in SCLC, remains a hallmark genomic feature in C-SCLC, with reported inactivation rates exceeding 50% in C-SCLC (8–10). Notably, the unique histological heterogeneity within C-SCLC raises critical questions regarding its distinctive tumor evolution trajectories, metastatic patterns, and resistance mechanisms to systemic therapies (11, 12). This biological complexity underscores the urgent need for clinicopathological studies to stratify patients for precisely tailored therapeutic interventions and optimize long-term oncologic outcomes. However, the optimal therapeutic approach for C-SCLC has not been fully established. It is often aligned with the conventional SCLC treatment guidelines and a multidisciplinary comprehensive approach. According to current evidence, C-SCLC patients typically receive multimodal therapy, including surgery, chemotherapy, and radiotherapy (13). Since the Food and Drug Administration’s (FDA) approval of atezolizumab for the first-line treatment of extensive-stage SCLC (ES-SCLC) within the landmark Impower133 study in 2019, the therapeutic landscape for SCLC has entered the immunotherapy era (14, 15). Immune checkpoint inhibitors (ICIs) combined with etoposide-platinum (EP) chemotherapy are recommended as the preferred approach for ES-SCLC patients. However, data on immunotherapy for C-SCLC patients are limited, with only a few case report studies (16, 17).

In this retrospective, real-world research, our objective was to describe the clinical and pathological characteristics of C-SCLC patients and investigate their treatment modalities and prognosis in real-world clinical practice. Furthermore, to contextualize our findings within the broader landscape of C-SCLC research, we conducted a systematic literature review summarizing existing evidence on C-SCLC epidemiology, molecular biology, treatment paradigms, and clinical outcomes. This integrated approach will provide a more comprehensive understanding of C-SCLC and inform future treatment strategies.

## 2 Patients and methods

### 2.1 Study design and study population

This observational retrospective study was conducted by utilizing the institutional electronic medical records system at Sir Run Run Hospital. We systematically reviewed the medical records of patients with a clinical diagnosis of lung cancer who presented at our hospital from January 2017 to December 2024, with the final follow-up completed by December 2024. Cases with histopathologically confirmed C-SCLC were included in this analysis. The diagnostic criteria of C-SCLC according to the NCCN guidelines for SCLC (version 2. 2022) were as follows (4): C-SCLC consists of both SCLC histology and NSCLC histology (squamous cell, adenocarcinoma, spindle/pleomorphic, and/or large cell carcinoma). No minimal percentage of NSCLC histologic elements is required for the classification of combined SCLC; if any elements are present along with SCLC, then this can be classified as combined SCLC. The exception is when SCLC is combined with LCNEC. At least 10% of the tumor should show LCNEC morphology to be classified as combined SCLC and LCNEC. The other specific inclusion criteria were patients aged 18 years and above who were receiving anti-tumor systemic treatment without particular limitations on the treatment regimen. Exclusion criteria were: (1) patients with insufficient data; (2) patients combined with other primary malignant tumors; (3) for patients who received immunotherapy, those with any active autoimmune disease, a history of autoimmune disease, or those who have been on long-term or high-dose use of corticosteroids or other immunomodulatory agents should be excluded.

This study protocol was reviewed and approved by the Ethics Committee of Sir Run Run Hospital (ethical approval number: 2025 - 0342), and written informed consent from patients was waived due to the retrospective study design. All patient data were anonymized and handled in compliance with the Declaration of Helsinki and reported following the Strengthening the Reporting of Observational Studies in Epidemiology (STROBE) guidelines (**Supplementary File**).

### 2.2 Data and assessments

Two independent researchers (Liu Gong and Hongseng Li) conducted blinded medical record abstraction according to a standardized study protocol, and any inconsistencies in judgment were discussed and adjudicated by a third investigator (Jiawei Shou). Data collection encompassed both structured variables (such as age and gender) and unstructured textual data (such as diagnosis, medical history, general condition, and disease course records). Utilizing a pre-validated electronic case report form (eCRF), the researchers systematically extracted the demographic and clinicopathological characteristics at the initially diagnosed stage, including gender, age, family history of tumor, tobacco exposure, comorbidities, pathological diagnosis, clinical stage, metastasis status, Eastern Cooperative Oncology Group performance status (ECOG PS), and driver gene alteration status. The researchers also recorded the details of patients’ treatment, including surgery, radiotherapy, systemic treatment status (including perioperative and advanced disease treatment options), and progression/recurrence patterns.

The efficacy was assessed utilizing the progression-free survival (PFS), tumor response rate (RR), and disease control rate (DCR). PFS was defined as the time from the first dose of treatment to the first documented disease progression or death of any cause. The RR and DCR were assessed in patients with measurable disease at baseline and received at least one imaging evaluation after treatment. The tumor response was evaluated by investigators according to the Response Evaluation Criteria in Solid Tumors version 1.1 (RECIST v1.1). RR is defined as the proportion of patients who achieve a complete response (CR) or partial response (PR); DCR is the proportion of patients who achieve CR, PR, or stable disease (SD).

### 2.3 Statistical analysis

The Shapiro-Wilk test was used to assess the normality of continuous variables. According to the normality test results, continuous variables were described as mean ± standard deviation (SD) or median (range). Categorical variables were summarized as counts and percentages. The 95% confidence intervals (CIs) for RR and DCR were calculated using the Clopper-Pearson method. The median PFS with corresponding 95% CI was calculated utilizing the Kaplan-Meier method. Subgroup survival analyses were stratified by different clinicopathological characteristics and treatment regimens, and hazard ratios with corresponding 95% CIs were displayed. Statistical analyses were performed with the R software (version 4.3.2), and a two-sided *P*-value < 0.05 was considered statistically significant.

## 3 Results

### 3.1 Clinicopathological characteristics

**Figure 1:**
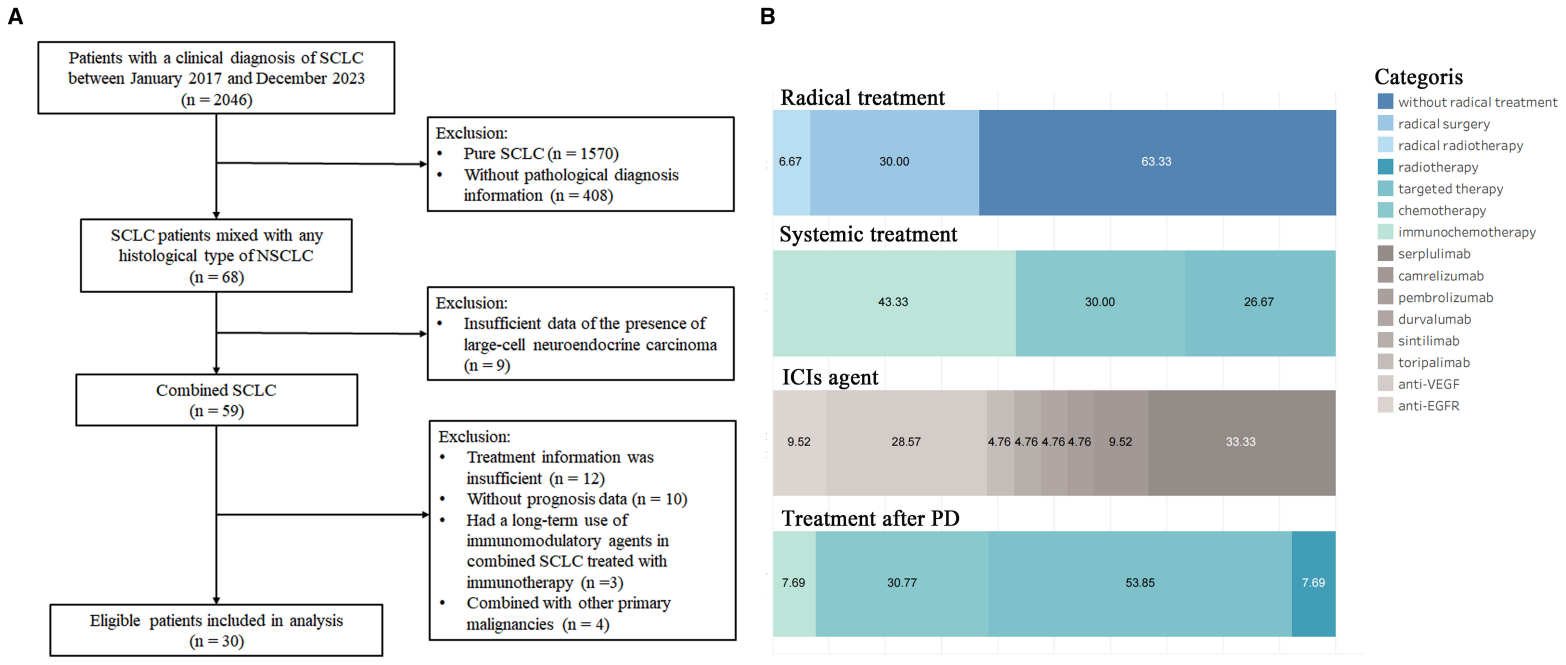
**(A)** Flowchart of patient selection. **(B)** Treatment modalities of included patients.

From January 2017 to December 2024, 59 patients with C-SCLC were included in the patient screening process. Based on predefined inclusion/exclusion criteria, 30 patients were ultimately enrolled (**Figure 1A**). Baseline clinicopathological characteristics are summarized in **Table 1**. The cohort had a mean age of 63.77 ± 9.28 years, with 14 patients (46.67%) aged 65 and above. Most participants were male (n=25, 83.33%), non-smokers (n=20, 66.67%), had stage IV disease (n=23, 76.67%), and exhibited an ECOG PS of 1 (n=30, 100.00%). No pulmonary comorbidities were reported, while hypertension was present in 10 patients (33.33%). Lymph node metastasis occurred in 27 cases (90.00%), contralateral lung metastasis in 13 patients (43.33%), and metastases to the liver (n=3, 10.00%), brain (n=3, 10.00%) and bone (n=2, 6.67%) were documented in a minority of cases. Histopathological analysis revealed the most common subtypes to be SCLC combined with AC (SCLC/AC; n=11, 36.67%) and SCC (SCLC/SCC; n=11, 36.67%), followed by SCLC combined with LCNEC (SCLC/LCNEC; n=8, 26.67%).

**Table 1:** Clinicopathological characteristics and treatments of included C-SCLC patients (N = 30).

Characteristics, n (%)	Total (N = 30)
Age (years), mean ± SD	63.77 ± 9.28
Age (years)	
< 65	16 (53.33)
≥ 65	14 (46.67)
Gender	
Male	25 (83.33)
Female	5 (16.67)
Smoking	
Current/Former	10 (33.33)
No	20 (66.67)
Family cancer history	
No	30 (100.00)
ECOG PS	
1	30 (100.00)
Comorbidities	
Pulmonary disease	0 (0.00)
Hypertension	10 (33.33)
Clinical stage	
IIB	1 (3.33)
IIIA	1 (3.33)
IIIB	2 (6.67)
IIIC	3 (10.00)
IV	23 (76.67)
Mixed tumor histology	
SCC	11 (36.67)
AC	11 (36.67)
LCNEC	8 (26.67)
Metastasis site	
Lymph node	27 (90.00)
Contralateral lung	13 (43.33)
Liver	3 (10.00)
Brain	3 (10.00)
Bone	2 (6.67)
Other	8 (26.67)
Previous radical treatment	
Surgery	9 (30.00)
adjuvant chemotherapy	7 (23.33)
consolidation radiotherapy	3 (10.00)
Radical radiotherapy	2 (6.67)
radiochemotherapy	2 (6.67)
immune consolidation	2 (6.67)
Systemic treatment modality	
Immunochemotherapy ^a^	13 (43.33)
Chemotherapy ^b^	9 (30.00)
Targeted therapy ^c^	8 (26.67)
Treatment after progression	
Radiotherapy ^d^	1 (3.33)
Immunochemotherapy	1 (3.33)
Chemotherapy	4 (13.33)
Targeted therapy ^e^	7 (23.33)

SD, standard deviation; ECOG PS, Eastern Cooperative Oncology Group performance status; SCC, squamous cell carcinoma; AC, adenocarcinoma; LCNEC, large-cell neuroendocrine carcinoma.

a Immune checkpoint inhibitors included serplulimab (n=7), camrelizumab (n=2), pembrolizumab (n=1), durvalumab (n=1), sintilimab (n=1), and toripalimab (n=1); platinum-based chemotherapy were used in the combination therapy.

b Platinum-based chemotherapy regimens were used.

c Targeted therapy included anti-EGFR-based therapy (osimertinib or icotinib; n=2), and anti-VEGF-based therapy (anlotinib or bevacizumab; n=6).

d The patient received whole-brain radiotherapy due to extensive brain metastases.

e Patients received anlotinib-based therapy (n=7).

### 3.2 Treatment

The details of the treatment are displayed in **Table 1** and **Figure 1B**. Among the 30 patients, 11 patients (36.67%) had received radical treatment, with nine receiving surgical resection and two receiving radical radiotherapy. Of the nine surgical patients, seven received adjuvant chemotherapy, and three underwent consolidation radiotherapy; two patients were treated with concurrent radiochemotherapy followed by immunotherapy consolidation. For systemic treatment strategies, 13 patients (43.33%) were treated with ICIs combined with platinum-based chemotherapy. The ICIs included serplulimab (n=7), camrelizumab (n=2), pembrolizumab (n=1), durvalumab (n=1), sintilimab (n=1), and toripalimab (n=1). Nine patients (30.00%) were treated with platinum-based chemotherapy alone, while the remaining cases (n=8, 26.67%) were managed with antiangiogenic therapies (anlotinib or bevacizumab; n=6) or anti-EGFR therapies (osimertinib or icotinib; n=2). Additionally, 13 out of 18 patients who experienced progressive disease received treatment after the disease progression. The treatments included anlotinib-based targeted therapy (n=7), platinum-based chemotherapy (n=4), immunochemotherapy (n=1), and one patient received whole-brain radiotherapy due to extensive brain metastases.

### 3.3 Prognosis

Among the 27 patients with measurable disease, two (7.41%) had CR, 12 (44.44%) had PR, nine (33.33%) had SD, and four (14.81%) had progressive disease following systemic treatment; two patients had target lesions disappear after receiving serplulimab-based immunochemotherapy. One was SCLC/SCC and the other was SCLC/LCNEC. The ORR was 51.85% (14/27; 95% CI: 31.95% - 71.33%), and DCR was 85.19% (23/27; 95% CI: 66.27% - 95.81%), respectively.

**Figure 2:**
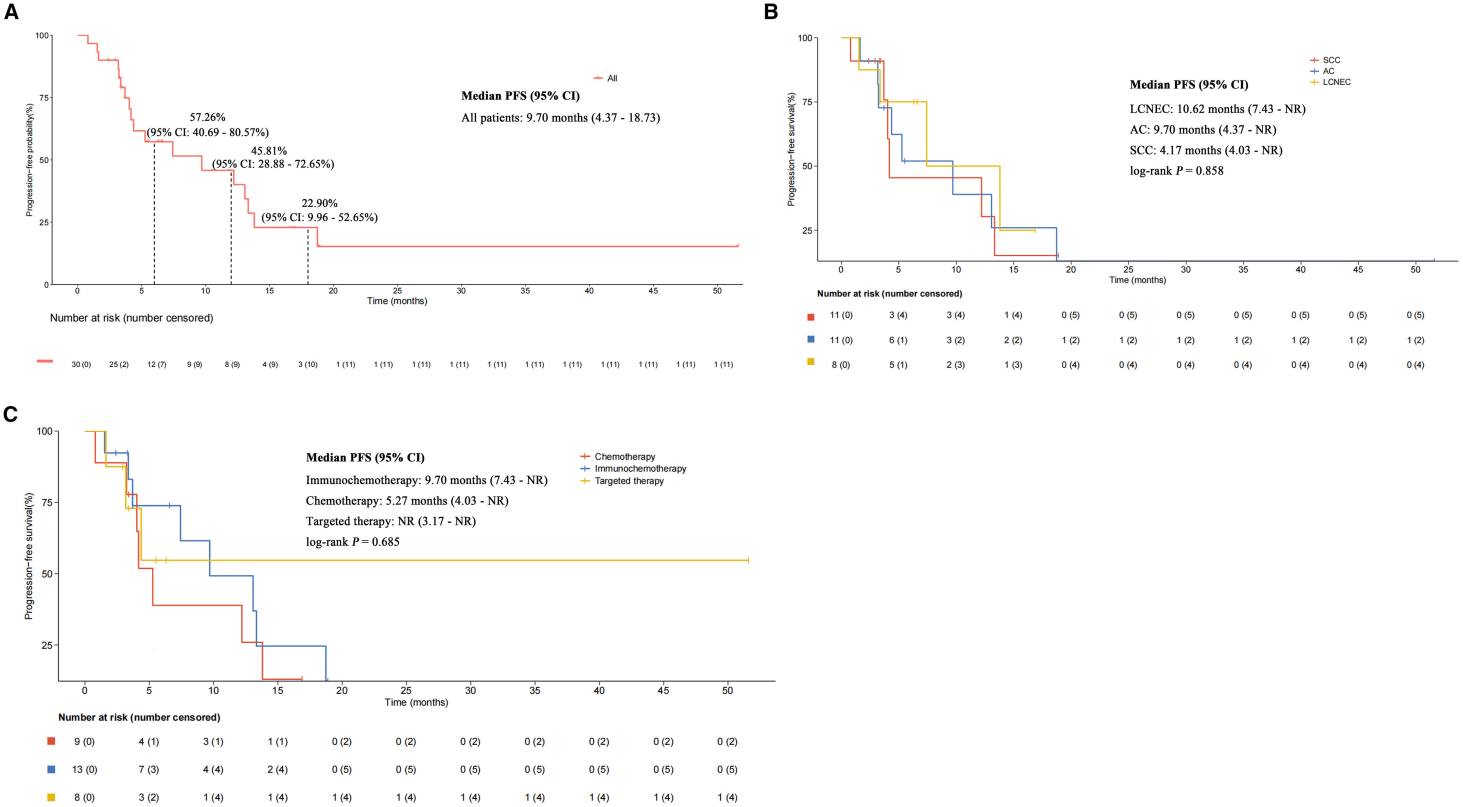
Kaplan-Meier estimates of progression-free survival. **(A)** All patients. **(B)** Subgroup analysis stratified by different mixed histological types. **(C)** Subgroup analysis stratified by different treatment strategies.

The median follow-up duration was 9.66 months. Overall, 18 patients (60.00%) experienced PFS events, with a median PFS of 9.70 months (95% CI: 4.37 - 18.73) and a 6-month PFS rate of 57.26% (95% CI: 40.69% - 80.57%) (**Figure 2A**). According to the subgroup analysis of different NSCLC histological types, a poor median PFS could be observed in SCLC/SCC patients (4.17 months; 95% CI: 4.03 - NR), compared with SCLC/AC (9.70 months; 95% CI: 4.37 - NR) and SCLC/LCNEC (10.62 months; 95% CI: 7.43 - NR), with a log-rank *P*-value of 0.858 (**Figure 2B**). In the subgroup analysis stratified by different treatment regimens (**Figure 2C**), the median PFS of patients who received immunochemotherapy (9.70 months; 95% CI: 7.43 - NR) was higher than that of patients treated with chemotherapy (5.27 months; 95% CI: 4.03 - NR) and targeted therapy (NR; 95% CI: 3.17 - NR), although the difference between groups was not statistically significant (log-rank *P-*value = 0.685). We further explored the associations between PFS and age, gender, smoking status, clinical stage, metastatic patterns, and prior radical treatment. While statistical significance was not reached, trends toward prolonged PFS were observed in the following subgroup: patients aged ≥ 65 years (9.70 *vs.* 7.43 months; *P* = 0.485), female (18.73 *vs.* 7.43 months; *P* = 0.641); non-smokers (9.70 *vs.* 4.37 months; *P* = 0.707), those without stage IV disease (13.80 *vs.* 5.27 months; *P* = 0.114), those with absence of lymph node metastasis (18.73 *vs.* 7.43 months; *P* = 0.351), those with absence of contralateral lung metastasis (12.20 *vs.* 4.37 months; *P* = 0.499), and those undergoing radical treatment (9.70 *vs.* 7.43 months; *P* = 0.619). See **Table 2**.

**Table 2:** Subgroup analysis of PFS according to different clinicopathological characteristics and treatments.

Characteristics	Sample size (N = 30)	Median PFS, months (95% CI)	log-rank *P*	HR (95% CI)	*P*
Age (years)					
< 65	16	7.43 (4.17 - NR)	0.483	Ref.	
≥ 65	14	9.70 (4.03 - NR)		0.72 (0.28 - 1.83)	0.485
Gender					
Male	25	7.43 (4.17 - 13.80)	0.640	Ref.	
Female	5	18.73 (NR – NR)		0.70 (0.16 - 3.10)	0.641
Smoking					
No	20	9.70 (4.17 – NR)	0.707	Ref.	
Current/Former	10	4.37 (3.70 – NR)		1.22 (0.43 – 3.49)	0.707
Clinical stage					
II/III	7	13.80 (9.70 - NR)	0.100	Ref.	
IV	23	5.27 (4.03 - NR)		2.76 (0.78 - 9.71)	0.114
Mixed tumor histology					
SCC	11	4.17 (4.03 - NR)	0.858	Ref.	
AC	11	9.70 (4.37 - NR)		0.97 (0.34 - 2.83)	0.962
LCNEC	8	10.62 (7.43 - NR)		0.72 (0.20 - 2.58)	0.613
Lymph node metastasis					
No	3	18.73 (NR - NR)	0.333	Ref.	
Yes	27	7.43 (4.17 - 13.80)		2.63 (0.34 - 20.08)	0.351
Contralateral lung metastasis					
No	17	12.20 (7.43 - NR)	0.497	Ref.	
Yes	13	4.37 (4.03 - NR)		1.39 (0.54 - 3.56)	0.499
Previous radical treatment					
No	19	7.43 (4.37 - NR)	0.554	Ref.	
Yes	11	9.70 (3.37 - NR)		0.73 (0.21 - 2.54)	0.619
Systemic treatment modality					
Chemotherapy	9	5.27 (4.03 - NR)	0.685	Ref.	
Immunochemotherapy	13	9.70 (7.43 - NR)		0.69 (0.25 - 1.95)	0.490
Targeted therapy	8	NR (3.17 - NR)		0.59 (0.15 - 2.33)	0.449

PFS, progression-free survival; HR, hazard ratio; CI, confidence interval; SCC, squamous cell carcinoma; AC, adenocarcinoma; LCNEC, large-cell neuroendocrine carcinoma; NR, not reached.

## 4 Discussion

The present study provides a single-center, retrospective analysis of clinicopathological characteristics, treatment patterns, and survival outcomes in 30 C-SCLC patients. Our findings highlight their distinct epidemiological profiles and prognostic heterogeneities among histological subtypes and treatment regimens, which could provide helpful stratifications for clinical decision-making of systemic treatment in C-SCLC.

Divergent specimen collection methodologies in various medical institutions have led to substantial prevalence fluctuations (2% - 30.1%) for C-SCLC [3 - 5]. As reported by Fushimi and his colleagues (18), the frequency of C-SCLC in the primary sites was statistically higher in autopsy specimens than in biopsy or cytology specimens (14.3% *vs.* 8.6%; *P* < 0.05). Furthermore, while advances in screening tools and diagnostic techniques have improved C-SCLC identification, the accurate subclassification of C-SCLC remains challenging in small biopsy specimens due to an increase in extrusion artefacts, making it necessary to rely on bronchoscopy and needle aspiration biopsy (19, 20). Contrary to the well-documented association between lung cancer and heavy smoking (21, 22), most patients in our cohort were without tobacco exposure. This observation may have two explanations. Firstly, this study was a single-center retrospective analysis, which is inherently limited by a relatively small sample size that may introduce potential biases. In addition, this discrepancy may reflect inherent biological differences in C-SCLC carcinogenesis, potentially involving alternative oncogenic pathways such as EGFR mutations or ALK rearrangements more prevalent in non-smokers and those with AC (23, 24). Historical data show that EGFR mutations are rare in pure SCLC, occurring in less than 5% of cases, but the prevalence increases to 15% - 20% in C-SCLC (25–27). Lei et al. (28) identified LCNEC as the predominant histologic subtype in C-SCLC; Men et al. (29) found SCC was the most frequent component; most SCLC patients in our study were mixed with AC or SCC. This inter-study heterogeneity may be attributed to various uncontrollable factors, such as the gender ratio, smoking history, and individual genetics.

The therapeutic landscape of C-SCLC remains challenging due to its intrinsic biological complexity. A central obstacle lies in the profound tumor heterogeneity of C-SCLC, where coexisting small cell and non-small cell components exhibit divergent molecular profiles and therapeutic vulnerabilities. For example, the SCLC component typically harbors bi-allelic inactivation of *RB_1_*/*TP_53_* and demonstrates sensitivity to platinum-based chemotherapy (10, 30); the NSCLC components may retain oncogenic drivers such as EGFR mutations or ALK rearrangements (31, 32). This genomic bifurcation creates a therapeutic dilemma that conventional chemotherapy effectively targets the rapidly proliferating SCLC clones but exerts limited control over NSCLC subpopulations, while molecularly targeted agents, though validated in NSCLC, often fail against SCLC-dominant tumors due to intrinsic resistance mechanisms. Despite these challenges, the preferred treatment options remain unclear, and the common approach is to follow conventional SCLC treatment guidelines. Surgical therapy may be crucial for patients with early-stage disease, and immunotherapy, chemotherapy, and targeted therapy should be considered for advanced disease (13). In our study, most patients were treated with a combination of immunotherapy and chemotherapy, which demonstrated a numerically superior survival benefit compared with chemotherapy and targeted therapy (median PFS: 9.70 *vs.* 5.27 *vs.* NR months). This finding aligns with recent phase III trials demonstrating the survival benefits of ICIs combined with EP regimen compared with chemotherapy in patients with ES-SCLC (33, 34). However, the evidence of immunochemotherapy in C-SCLC patients is limited. Theoretically, C-SCLC is anticipated to be more susceptible to immunotherapy, given the highly unstable nature of the genome and chromosomes in SCLC. Liu et al. (35) reported that an SCLC patient with lung squamous cell carcinoma (LUSC) and high TMB achieved sustained clinical benefit from anti-PD-1 inhibitor therapy as a third-line treatment, with a PFS of 9.7 months. Qu et al. (36) documented a case initially diagnosed with SCLC that progressed to SCLC/AC after first-/second-line chemotherapy and radiotherapy. The patient showed stable lung lesions following third-line treatment with pembrolizumab, indicating a potential advantage of immunotherapy. In our cohort, six patients were treated with antiangiogenic therapies (anlotinib or bevacizumab) in the frontline, and seven patients received anlotinib after disease progression, which aligns with clinical practice guidelines. Anlotinib is recommended as a third-line treatment option for patients with SCLC in Chinese clinical practice, which can significantly prolong PFS by 3.4 months and reduce the risk of disease progression by 81% (37, 38). Recently, a phase III trial demonstrated that immunochemotherapy combined with anlotinib as first-line therapy could result in significant survival benefits for ES-SCLC compared to placebo plus chemotherapy (39). Furthermore, two patients with SCLC/AC patients received EGFR-TKIs (osimertinib and icotinib) but had a relatively limited prognosis, with a PFS of 1.63 and 3.17 months, respectively. Although EGFR-TKIs are widely used in NSCLC patients with EGFR mutations, the efficacy of EGFR-TKIs may vary in C-SCLC. Takagi et al. (40) reported a case with EGFR L861Q mutation in both SCLC and AC components, in which multiple brain metastases and enlarged mediastinal lymph nodes subsequently appeared after second-line erlotinib treatment. Another study reported a woman with SCLC/AC with an L858R mutation who achieved PR after gefitinib treatment (25). While EGFR-TKIs might be applied to C-SCLC harboring EGFR mutations, the limited data available makes it difficult to precisely determine their efficacy, which may also be less pronounced in SCLC or C-SCLC than in NSCLC.

Additionally, prognostic heterogeneity was found among histological subtypes in our study. The observed PFS gradient across subtypes (SCC/LCNEC: 10.62 *vs.* SCLC/AC: 9.70 *vs.* SCLC/SCC: 4.17 months) reveals clinically meaningful biological diversity. A retrospective analysis of 181 stage I-IIIa C-SCLC who received radical R0 surgery and platinum-based chemotherapy indicated that SCLC/LCNEC patients had a better prognosis compared with SCLC/AC and SCLC/SCC, with a median disease-free survival (DFS) of 44.1 *vs.* 20.4 months (*P* = 0.040) (28). The better prognosis in SCC/LCNEC may relate to preserved neuroendocrine differentiation and pathway genes of SCLC (*TP_53_*/*RB_1_*) and NSCLC (*STK_11_*/*KEAP_1_*/*RAS*), potentially enhancing sensitivity to platinum-based regimens (41). This finding suggests that molecular profiling in C-SCLC may guide personalized management for different patient groups. In addition, the prognosis of C-SCLC is modulated by multiple clinicopathological and treatment-related factors. In a population-based retrospective analysis of 784 C-SCLC cases identified from the SEER database between 2004 - 2016, researchers found that patients with poor differentiation and stage IV disease had worse survival (42). Another analysis of 114 cases with C-SCLC identified smoking, Karnofsky performance score (KPS) < 80, advanced TNM stage, no surgery, positive resection margin, positive lymph nodes ≥ 4, positive lymph node ratio > 10%, and non-multimodality treatment as risk factors for poor OS (29). Notably, the prognostic significance of emerging biomarkers, such as PD-L1 expression levels and TMB, remains undefined, highlighting a critical gap in precision prognostication for this heterogeneous malignancy.

Although our study provides novel insights into immunochemotherapy and targeted therapy in C-SCLC, this study has several limitations. Firstly, due to the lower incidence and difficulty in diagnosis, a small sample size was included in our study, which introduces potential selection bias and limits the generalizability of our findings to the broader C-SCLC population. While this study reveals distinct PFS patterns among C-SCLC histological subtypes, the subgroup comparisons were inherently limited by cohort size, preventing adjustment for clinically relevant confounders, including disease stage, performance status, and therapeutic heterogeneity. These unadjusted analyses must be interpreted with caution, requiring rigorous validation in future dedicated cohorts with sufficient power for robust statistical adjustment. Additionally, this single-center, retrospective study design limits the ability to establish causative relationships between treatment regimens and outcomes. Lastly, the limited genetic testing and lack of comprehensive analysis of driver genes and biomarkers prevent us from fully exploring the underlying mechanisms and identifying patients who may derive the greatest benefit from different therapy options.

## 5 Conclusion

In our cohort, SCLC/AC and SCLC/SCC were the most common subtypes of C-SCLC, but patients with SCLC/LCNEC showed longer survival than those with other mixed histopathological types. Immunochemotherapy was the primary treatment regimen for C-SCLC, and our data suggest that patients are more likely to benefit from this approach. Our findings suggest the need to further explore the relationship between different histopathological types and prognosis, and the investigation of biomarker-driven patient selection may facilitate the identification of optimal therapeutic strategies for patients with C-SCLC. To definitively validate these subtype-specific survival patterns and therapeutic implications, multi-institutional collaborative efforts are warranted to establish evidence-based precision frameworks.

## Data Availability

The original contributions presented in the study are included in the article/**Supplementary Material**. Further inquiries can be directed to the corresponding author.
